# Family caregiver quality of life and the care provided to older people living with dementia: qualitative analyses of caregiver interviews

**DOI:** 10.1186/s12877-022-02787-0

**Published:** 2022-01-31

**Authors:** Afeez Abiola Hazzan, Jason Dauenhauer, Patti Follansbee, Joyce O Hazzan, Katy Allen, Ibukun Omobepade

**Affiliations:** 1 Department of Healthcare Studies, the College at Brockport, State University of New York, 350 New Campus Drive, Brockport, NY 14420 USA; 2 Department of Social Work, the College at Brockport, State University of New York, 350 New Campus Drive, Brockport, NY 14420 USA; 3 School of Business, Liberty University, 1971 University Blvd, Lynchburg, VA 24515 USA; 4 Lifespan of Greater Rochester, Inc, 1900 S. Clinton Avenue, Rochester, NY 14618 USA; 5 Department of Criminal Justice and Security, Pace University, One Place Plaza, New York, NY 10038 USA

**Keywords:** Aging, Family caregivers, Dementia, Quality of life, Quality of care, Level of care, Alzheimer’s disease, Social support, COVID-19 pandemic

## Abstract

**Background:**

While studies have examined the quality of life (QoL) of family caregivers for people living with dementia, it is not yet clear how these caregivers’ quality of life affects their ability to meet the care demands of their loved ones. The purpose of the study was to explore caregivers’ perceived quality of life in relation with the care provided to persons with dementia.

**Methods:**

Twenty-three caregivers participated in this study. These caregivers participated in focus groups or one-on-one interviews after completing questionnaires regarding their own quality of life as well as the care provided to their loved ones with dementia. The interview and focus group transcripts were analyzed using qualitative description methodology.

**Results:**

Regarding the relationship between caregiver QoL and the care provided to older people with dementia, the following themes were derived: (1) needs of the person with dementia; (2) caregivers’ ability to cope with progressing needs of the care recipient; (3) availability and accessibility of support for the caregiver; (4) unmet needs of the caregiver; and (5) ability of the caregiver to meet their own progressing needs as the condition of the care recipient deteriorates.

**Conclusions:**

Findings from this study suggest that caregiver quality of life is important for the care they provide to their loved ones living with dementia. The study highlights the need for the provision of much needed support for caregivers and older people living with dementia. These issues are particularly important during and post-pandemic.

## Background

Due to rapid population aging and other factors, the global prevalence of dementia is growing [1]. Approximately 50 million people are currently living with dementia worldwide, and this number is projected to reach 152 million people by 2050 [1, 2]. Estimates indicate 5.7 million people are currently living with dementia in the United States, and the numbers are projected to rise as population aging continues [3]. While several types of dementia have been identified, Alzheimer’s disease (AD) accounts for 60% to 70% of all cases [1]. By 2050, approximately 13.8 million people in the United States are projected to have Alzheimer’s disease [3].

Family caregivers of older people living with dementia are relatives, friends, or neighbors who provide assistance related to this condition, but who are unpaid for the services they provide [1, 4]. These caregivers play a critical but often overlooked role in meeting the needs of older adults who have dementia. They help perform many duties for their loved ones including shopping for groceries, helping with medications, managing finances and legal affairs, guarding against wandering, and helping with basic and instrumental activities of daily living [4].

Importantly, the caregiving role becomes more demanding as the disease progresses over time, and studies have shown that the quality of life (QoL) of caregivers for persons living with dementia is lower than the QoL of caregivers for persons who do not have this disease [5]. Compared to family caregivers of people with other conditions like cancer, family caregivers of older people living with dementia often suffer higher levels of burden and depression [6].

In a qualitative study comparing the views of people with dementia and family carers regarding the quality of end-of-life care, the authors identified several key consideration including the need for receiving care in place and ensuring comfort for the care recipient [7]. In addition, a review and thematic synthesis of qualitative studies examining the perspectives of people with dementia and their carers on advance care planning and end-of-life care identified five key themes [8]. These themes are: avoiding dehumanizing treatment and care; confronting emotionally difficult conversations; navigating existential tensions; defining personal autonomy; and lacking confidence in healthcare settings. In another qualitative study exploring the views of family caregivers about the quality of end-of-life care for people living with dementia, the authors identified three inter-related themes including maintaining the person within, fostering respect and dignity, as well as showing compassion and kindness [9].

In addition, a multi-method qualitative study with key stakeholders including people with dementia and their family caregivers, sought to answer the question “what enables good end-of-life care for people with dementia?” [10]. Thematic and integrative analysis of the data yielded seven key factors required for the delivery of good end-of-life care for people with dementia. These factors include: timely planning of discussions; recognition of end-of-life and provision of supportive care; co-ordination of care; effective working relationships with primary care; managing hospitalization; continuing care after death; and valuing staff and ongoing learning.

In a qualitative study investigating barriers to achieving high-quality, community-based integrated dementia care, the researchers identified nine key themes including: little attention given to patient wishes, lack of time and space to provide high-quality care, disturbing hospital environment, poor compensation for staff members, refusing to visit outpatient clinics, declined admission, daily life support by family members and caregivers, dementia care team, and community bonding [11].

Further, a systematic meta-synthesis of fourteen qualitative studies describing the experiences, quality of life, and psychosocial impact of caregiving on 265 adult informal caregivers of people with dementia identified five themes [12]. The first theme concerned the need for caregivers to understand and make sense of dementia diagnosis, worsening symptoms, as well as their evolving role. Secondly, caregivers described coping strategies and rewards of caregiver role as being important for their quality of life. Also, challenges associated with caring for a person with dementia and managing their behavioral symptoms were identified as important factors that contribute to the psychosocial impact of caregiving on the caregiver. In addition, the quality of the caregivers' relationships with care-recipient and other informal caregivers affects the caregiver’s perception of caregiving, which in turn affect their quality of life. Finally, caregivers' experiences of formal support services and material resources play an important role in determining their experiences, quality of life, and the psychosocial impact of caregiving [12]. Overall, these findings point to a need for a person-centered approach to dementia care planning that recognizes the need for caregivers to experience better well-being and quality of life.

Although several studies have provided evidence for positive aspects of caregiving including lower depression and higher life satisfaction rate, the majority of caregivers find this role to be stressful [5, 6, 13–15]. In addition to the stressful nature of caregiving, the care recipient’s condition tends to decline over time, making the caregiving role more demanding with disease progression [6, 15]. This may in turn affect the caregiver’s ability to meet their loved ones’ care needs, resulting in a decline in the quality or level of care provided to the care recipient as the disease progresses.

However, there has been no research conducted to directly investigate whether lower caregiver QoL (both pre-existing QoL or changing QoL as a function of caregiving) affects the care (level or quality of care) that caregivers provide to persons with dementia. In a randomized controlled trial assessing the effectiveness of the STrAtegies for RelaTives (START) intervention in the short and long term, the researchers found that an eight-session manual-based coping intervention resulted in decreased anxiety and depression levels for caregivers [16]**.** Although the researchers also found evidence for improved QoL among caregivers, this did not result in improvement of the QoL of the person living with dementia. In a published systematic review examining the relationship between caregiver QoL and the quality of care provided [17], the researchers found only one study that was somewhat relevant [18]. This single study did not directly examine the association between QoL and level or quality of care provided to people with dementia. Rather, proxy measures of QoL (i.e. the Perceived Change Index) and quality of care (i.e. caregiver mastery) were utilized [17, 18].

Considering the lack of studies that have directly investigated the relationships between family caregiver QoL and the quality or level of care provided to persons living with dementia, it is necessary to explore this relationship. Results from such investigations could provide valuable information that would be useful to practitioners and stakeholders as society copes with the impact of caregiving for people with dementia. The purpose of the study was to explore family caregivers’ perceived quality of life in relation with the care provided to persons with dementia.

## Methods

### Study design and sampling

Each caregiver participated in a one-on-one or focus group in-person interview [19–21]. Table 1 presents a summary of the analytic process from raw data to theme interpretation. Purposeful sampling was used in selecting 23 caregivers to participate in focus groups or one-on-one interviews, depending on caregiver availability. Caregivers were recruited through caregiver serving organizations in the Rochester, New York area. Additional caregivers were recruited at a national caregiver conference in Chicago, Illinois. Recruitment took place between October 2018 and November 2019. Of the 23 caregivers who participated in this study, eight caregivers participated in two focus group sessions (four participants per group). Each caregiver participated in either a focus group or individual interview. Each focus group was led by the lead researcher (AH), with a student acting as a research assistant. Also, the lead researcher conducted all the one-on-one interviews. All focus groups and one-on-one interviews took place in the Rochester, New York area as well as in Chicago, Illinois. To be consistent with common research practice, caregivers of the person with dementia who provide uncompensated care for at least four hours per week were eligible for inclusion in this study [4, 5].

To commence recruitment, caregiver serving organizations forwarded information about the study to the caregivers currently receiving services from them. This included information regarding the purpose of the study as well as the inclusion criteria. Interested caregivers were advised to contact our research team to learn more about the study. Reminder notices were sent to each caregiver before the scheduled interview to remind them about the day, time, and location of the interview. Recruitment of additional caregivers ceased once data saturation had been reached [22, 23].

### Data collection and analysis

At the beginning of each interview session, caregivers completed a demographic questionnaire. The demographic questionnaire includes questions about the caregiver’s age, gender, education, relationship to care recipient, ethnicity, race, and current household income. A semi-structured interview guide was utilized during the interviews to raise pertinent open-ended questions for the participants to address. These questions included: (1) *could you please tell me about your quality of life?;* (2) *please tell me about the types of care you provide;* (3) *what does quality of care mean to you?;* (4) *what are the reasons why you are sometimes unable to meet your loved ones’ care needs?;* (5) *how are these reasons related to your quality of life as a family caregiver?*

The focus groups and one-on-one interviews took place between October 22, 2018 and November 10, 2019. The focus group interviews lasted for an average 68 min, and the one–one- one interviews lasted between 24 and 59 min. Data from the focus groups and interviews were transcribed verbatim using Otter [24]. The accuracy of the interview transcripts generated by Otter were verified by the first author (AAH).

Transcripts were analyzed using qualitative description methodology, which made it possible to summarize large amounts of text into themes or categories that represent similar meanings [22]. As described by Sandelowski (2010), qualitative description methodology facilitates the generation of findings that are closer to the data as given by the study participants [22]. This methodological approach is also useful for obtaining information that would be of use to practitioners and stakeholders dealing with a phenomenon.

Two authors (AAH and JD) independently read all the interview transcripts to achieve robust understanding of the data. After completing the data immersion process, the authors performed independent coding by writing down words or codes from each interview that seemed to capture important thoughts or concepts. These authors then met to compare codes, finalize the initial coding scheme, and to sort the codes into emergent themes that represent the key findings from the interviews. The study team members compared the results of their individual analyses during each of the meetings. Disagreements in the analyses results were discussed until a consensus was reached [22, 23]. Other authors provided feedback on the data analyses.

### Study rigor

Several strategies were used to achieve reliability, validity, and generalizability of the study findings [25–27]. For example, memos were kept throughout the course of conducting this study, including the data analysis stage. Also, an audit trail of all decisions related to caregiver recruitment, data collection, analysis, and writing was kept. To achieve investigator triangulation, four investigators (AAH, JD, JH, and IO) reviewed the interview transcripts and final results. Also, both focus groups and one-on-one interviews were used for data collection. To reduce researcher bias, all research team members provided feedback on the analysis and study findings. Further, the authors met periodically to compare their coding of the results, and to ensure that the meaning of the analysis is the same between the coders [25–27].

## Results

### Family caregiver sample

Table 2 shows the profile of the 23 caregivers who were interviewed in Rochester NY and Chicago IL. These caregivers were typically older adults (Median age = 62.0 years; interquartile range = 16.0 years), female (n = 18), technical/community college or university educated (n = 23), a spouse or child of an older adult with dementia (n = 22), and identified their race as white (n = 22). The major themes from the study are described below:

**Table 1:**
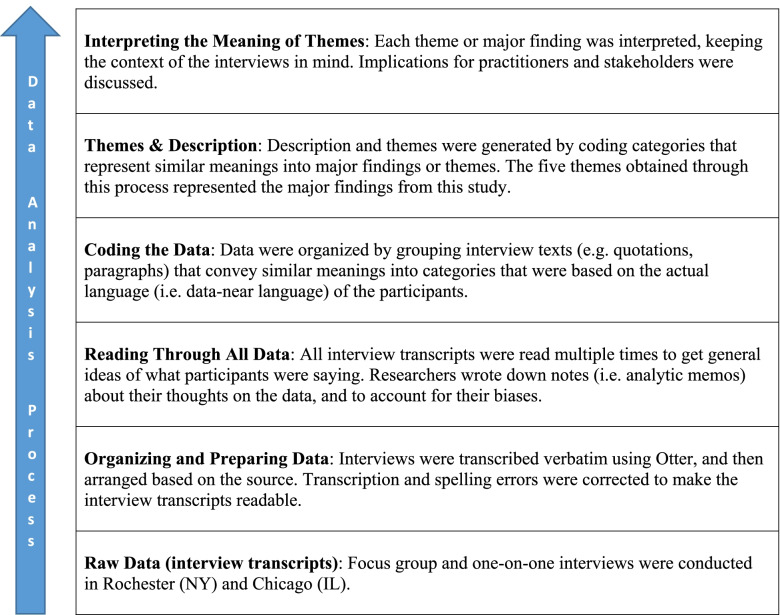
Summary of the data analysis process from raw data to theme interpretation

Adapted from Creswell & Creswell (2018) [19]

**Table 2:** Demographic characteristics of caregivers

**Characteristics**	**Caregivers**
Age in years, median (25–75 percentile; interquartile range)	62 (52, 68; 16)
Age range (years)	41–78
Gender, n (%)	
Women	18 (78.3)
Men	5 (21.7)
Education, n (%)	
Elementary school	0 (0)
High school	0 (0)
Technical/community college	2 (8.7)
College/University	21 (91.3)
Relationship to care recipient, n (%)	
Spouse	10 (43.5)
Child	12 (52.2)
Other	1 (4.3)
Ethnicity, n (%)	
Hispanic or Latino	1 (4.3)
Non-Hispanic	22 (95.7)
Race, n (%)	
Asian	1 (4.3)
White	22 (95.7)
Current annual household income, n (%)	
$0–$15,000	0 (0)
$15,001–$30,000	1 (4.3)
$30,001–$45,000	1 (4.3)
$45,001-$60,000	4 (17.4)
$60,001-$75,000	2 (8.7)
$75,000 or more	14 (60.9)
Missing	1 (4.3)

### Family caregiver quality of life and care provided

When considering how caregivers describe the relationship between their QoL and the care provided to their family members with dementia, analysis of the interviews revealed the following inter-related factors or themes: (1) needs of the person with dementia; (2) caregivers’ ability to cope with progressing needs of the care recipient; (3) availability and accessibility of support for the caregiver; (4) unmet needs of the caregiver; and (5) ability of the caregiver to meet their own progressing needs as the condition of the care recipient deteriorates.

#### Needs of the person with dementia

Each caregiver described a range of complex needs for their relative with dementia which seems to correspond to the progressive nature of the disease. As care recipients lose the ability to take care of themselves, these responsibilities are taken on by the caregiver. The following excerpt is from a female spousal caregiver who describes the current needs of her husband who has lost the ability to complete many activities of daily living on his own since his diagnosis of AD six years prior:

*My husband is no longer driving. And I'm the number one transporter. He's no longer taking his own medicines due to double dosing himself. And so I become a pharmacist. He's no longer able to make any of his own meals. He's not able to operate a microwave. And so I'm chief cook and bottle washer. (focus group)*

As a daughter, this caregiver has stopped working as an attorney to try to meet her mother’s needs:

*She is pretty physically independent, but just that she has no short-term memory and she can't remember to take pills and her sense of time is off so someone has to be there to sort of help her get ready to go somewhere. She can't drive so I drive her everywhere and just managing her doctor's appointments and I am her primary source of entertainment because she doesn't do a lot with her friends anymore. And I don't feel that I'm adequately meeting her needs because I think she needs to do more to get out more. And a lot of the times I just want to escape from her. (focus group)*

Caring for her husband with dementia associated with Parkinson’s disease, this female spouse describes having no quality of life due to her husband’s needs:

*I would say I have no quality of life because I have no life. 24/7, I need to be by my husband's side. If I need to leave my husband, I need to pay somebody to stay with him. So the little bit that I can afford each day, I get some help a few hours each day. But that means that all night long, every time he gets up to go to the bathroom, I need to be there right by his side to help him. Every time he moves from one chair to another or from the bed to a chair, or to the dinner table, I need to help feed him. He needs me by his side all the time. So I have no quality of life. I have no life other than being a full time aide to my husband. (one-on-one interview)*

In this final example, a daughter describes how she struggles to provide care for her mother who now resides in a nursing home and has progressing needs:

*So she's been there a year. My care of her is somewhere between 10 and 15 hours a week of direct care. I go once or twice a week, but I get phone calls from her sometimes up to 10 times a day in tears crying, just mixed up, can't find telephone numbers, doesn't get my phone calls and all that kind of stuff. But if I were to take on more of her care, my quality of life would be gone. (focus group)*

#### ***Caregivers’ ***ability*** to cope with progressing needs of the care recipient***

The caregivers who participated in this study were at different points along the caregiving journey. Many were doing their best to actively care for their family member at home with limited support, while others had reached, or were coming to the realization, they could no longer meet the needs of their relative and that professional caregiving services were necessary. All caregivers depicted a story of how their lives had changed to help meet the progressing needs of their care recipient. The following excerpt from a female spouse describes being overwhelmed due to the progression of responsibilities as a caregiver as her husband’s abilities decline. This is compounded by challenges related to parenting her teenage daughter and coping with the recent loss of her own parents. The caregiver explains that she is at a critical juncture in the caregiving journey, and is unable to provide quality care for her loved one:

*I've been executor of my parents’ estate…it's just been overwhelming. I'm now at a point where I'm considering stopping working because we are getting to the point where leaving him by himself for extended periods is probably not a good idea for much longer. So, it's very frustrating. As a caregiver, you get tired. You're juggling a lot of things mentally, physically, emotionally. And when you get tired, it's very difficult to juggle and be kind and caring for him. So, we get into arguments then. (one-on-one interview)*

Describing her husband’s disease progression and her limited ability to care for some of his needs, the following excerpt from a female spousal caregiver highlights the recognition of a future turning point in her caregiving journey:

*So I am starting to see that this is going to get harder and I'm not going to be able to meet his needs and he wants to stay home and I want him to stay at home. But at some point, I'm just not physically able to do what he needs me to do. (one-on-one interview)*

#### ***Availability and ****accessibility**** of support for the caregiver***

As indicated in the previous section, caregivers’ ability to cope with their relative’s progressing needs was often described in conjunction with ‘support’. Specifically, many participants described not having enough support to meet their care recipient’s needs. Caregivers often described coping with progressing needs by taking on more responsibilities until they could no longer function effectively and requiring external supports. This theme demonstrates how caregivers felt an often limited network of assistance affects their quality of life and the quality of care they provide. This first quote describes a daughter’s experience serving as the sole caregiver for her mother and a lack of support from other relatives:

*…So for the past year and a half, I don't really feel I exist anymore… I just don't feel I'm even a person anymore. I'm just basically making sure that she's living and, I just get through the day, every day…I don't have a quality of life…I believe that she needs to be in a facility right now, an assisted living…I can't do anymore…I can't continue to not exist and constantly struggle with trying to get her to take pills and eat and bathe…all these things and do it 100% alone. (focus group)*

Several caregivers displayed a positive attitude toward meeting their relatives’ needs. They described the importance of having a supportive network of family, friends, and professionals which helps them cope with their relatives’ progressing needs as evidenced by the following quote from a female spousal caregiver whose husband has Lewy body dementia:

*…And I take him to [a day program]… He goes there on Tuesdays for half a day. And I have a volunteer that comes once a week…I can't complain about support group. I have plenty of support. (one-on-one interview).*

Having support from her siblings and husband, this daughter describes having a good support system which has enhanced the quality of care provided. However, due to abuse allegations they are contemplating moving her parents from assisted living into their home:

*I have two siblings. All three of us are very involved with our parents. I have a husband who is actually on board with the possibility of moving them into our home. I can't say that that's not a stressor… I have a good strong supportive system for me. (one-on-one interview)*

Similarly, a male spouse describes a number of support services that have been helpful to him in providing quality care for his wife with dementia:

*Once the diagnosis happened, we were very engaged in various groups. We would go to seminars, we would go to all these drug trials which helped understand what was happening or at least give us a basis for such. We participated in couples support groups. I myself have been in a number of support groups in both either co-ed, or even a men's support group. I have engaged in counseling for myself to deal with anger management. (one-on-one interview)*

#### ***Unmet needs of ***the*** caregiver***

Most caregivers in this study reported sacrificing their own needs to help meet the physical, social, emotional and financial needs of the care recipient with dementia. All reported some level of unmet need when describing the effects of caregiving on their own quality of life and the quality of care they provide. These needs are often described as losses that negatively affect their own well-being, quality of life, and their ability to provide care.

Loss of companionship, loss of self, and unfulfilled plans are described by three different female caregivers describing the changing relationship with their husbands due to the progression of the disease:

*One biggest essential need that goes unmet is companionship and emotional connection. I know he still loves me, but it's just that I don't have that partner in the same way…I'd say that's an essential need that's not met. (one-on-one interview)*

*There’s a lot of grief that goes with that...And I don't get to talk to my spouse and have somebody say it's going to be okay. Because the person I would have gone to is now the person I can't go to. And I'm taking on all of his jobs, all of my jobs…there's just this intense grief of knowing that the future we plan together is not there anymore. And so in addition to all of the burden of caregiving, I also had the emotional burden of losing the person I thought I was going to spend the rest of my life with. (focus group)*

*We were planning on traveling after we retired….there's no way that he's going to be able to have the strength or the cognitive things that he needs…it was just going to be hard for both of us. (one-on-one interview)*

Unmet spiritual needs were reported by another female spouse. She discusses how she can’t meet her spiritual needs without paying someone to stay with her husband:

*Well, for example, one of my real needs is to be able to go to Mass on Sunday. I can't get there unless I hire somebody to stay with [my husband] so I can go to Mass. So, many times I'm not able to go to church… To me that's an essential need and it breaks my heart that I can't participate. (one-on-one interview)*

Lack of time, energy and being too tired to engage in exercise were reported by a number of caregivers as negatively affecting their ability to care for their loved ones. These are evidenced by the following three excerpts from female spousal caregivers:

*I'm pretty healthy. The thing that I don't get around to is exercise. I'd like to have more opportunity for that. Even times when I could take the opportunity, I'm too tired. (one-on-one interview)*

*I do not exercise, which is a major issue in that… I don't get enough sleep. All of these makes me constantly tired. All of these things, it's a fight to try to do those things. And they are majorly impacting you (one-on-one interview)*

*Before my husband got sick, I used to jog all the time. I love to jog. I haven't been able to do that in over a year and my chubby body is a proof of it… it's a question of time…you can't leave him alone… (focus group)*

Loss of friendships due to having little time to socialize was described by a few caregivers as affecting their quality of life and the care they provide. This is described in the following quote from a female spousal caregiver.

*..And it's hard we've lost most of our friends. We have one set of friends that still gets together with us… They've kind of given up on us…we tried to go out with two other friends and they didn’t think about the fact that there were steps to get up to the place where we are going to eat and it was hard for him… (one-on-one interview)*

Loss of freedom to make choices in comparison to those who don’t have caregiving responsibilities were described by this daughter who provides care for her mother.

*I feel most of my friends and family are able to come and go as they please and do what they need to do and take care of themselves and I don't have that option. I'm kind of trapped. (one-on-one interview)*

#### ***Ability of the caregiver to meet their own progressing needs as the condition of the care ***recipient*** deteriorates***

In this theme, caregivers describe a dynamic whereby the care recipients’ needs progress to the point where they are beyond, or at capacity for the caregiver’s control and abilities. This realization seems to be a turning point where caregivers may recognize their limitations in meeting their relatives’ needs. This recognition leads some caregivers to describe their progressive losses (social, emotional, physical, and financial) and to seek different types of support and to care for their own needs. However, others attempt to make more sacrifices by continuing to provide care until they reach that turning point.

The following example is from a female spouse caring for her husband with Parkinson’s related dementia:

*I would say that at the moment, I'm meeting all of his needs. But every day there is a decrease…it’s more progressive every day and there are more needs. And for me, I've reached my limit. Right now, we're managing but I've reached my limit.(one-on-one interview)*

Similarly, a male spouse articulates the importance of his own quality of life while recognizing his wife’s dementia will continue to progress.

*And I've come to accept the fact that her disease is not something I can control and that I have to focus on myself, my own quality of life, my own needs, my own personal needs, and set goals independent of [my wife] who will continue to deteriorate. (one-on-one interview)*

The decision to have her spouse transition to a nursing home is described by this female caregiver as a way to survive and maintain her health:

*And I decided I had to take care of myself…this was a big thing for me to be able to say out loud that I'm sorry, he's my husband, but I also have a priority. I hope I'm going to live longer than he does. And if my health is going to suffer from taking care of him, what good is that? That was a big realization to come to. (one-on-one interview)*

Trying to redefine oneself after being together with a partner for so many years is described by this female spouse in relation to her husband’s disease progression and impending death:

*And my husband is now into the late moderate stages. And I find myself practicing to be a widow. I'm trying to put myself in a position so that when he does die, I won't lose myself…I'm very blessed to have paid caregivers so that I do have some time to get out on my own and find those activities that are mine. At the same time, while I'm doing that, there's a sense of guilt, because he's not a part of that. And I'm having to look after myself and that's a hard thing to do after spending so many years of being ‘us’, and now learning to be ‘me’. (focus group)*

While a number of these previous excerpts highlight a turning point where the caregiver seems to be trying to meet their own needs by recognizing the limitations in their abilities, many participants were not at this point in their caregiving journey. The following example from a female spouse caring for her husband describes her intention of quitting her job to meet his needs. She is not yet focusing on her own needs or limitations even though the stress is overwhelming:

*I'm now at a point where I'm considering stopping working because we are getting to the point where leaving him by himself for extended periods is probably not a good idea for much longer. So, it's very frustrating. As a caregiver, you get tired. You're juggling a lot of things mentally, physically, emotionally. And when you get tired, it's very difficult to juggle and be kind and caring…* (one-on-one interview)

## Discussion

Results from this qualitative analyses of caregiver interviews and focus groups show that the relationship between caregiver quality of life and the care provided to older people with dementia is complex. Generally, caregivers of older people with dementia agree that the role offers both challenges and opportunities [5, 6, 13–15]. These are consistent with prior qualitative studies describing the ideal conditions for providing optimum care for older adults living with dementia, particularly in a community setting [7, 8, 11, 12]. To provide good quality of care, studies have shown that caregivers must pay attention to their loved one’s needs, devote adequate time and space for high quality care, and receive support to continue in their caregiving role [10, 11]. Also, it is important for caregivers to have access to formal support services and other resources that promote high quality of life [12]. Importantly, such support can come from both paid (for example, primary care or respite care) and unpaid (for example, other relatives) sources [10–12].

Considering the enormous challenges involved in dementia caregiving, most caregivers report doing the best they can for their relatives living with dementia [4, 15, 17]. As results from the current study show, caregivers recognize that there are several factors beyond their control that could make it impossible for them to always meet their loved ones’ needs. Among the reasons offered by participants in this study are lack of support and resources, especially for caregivers who have to juggle multiple tasks as part of their role.

As the needs of older adults with dementia increases over time due to declining health, the caregiver is often called upon to perform even more duties, unless they are willing to transfer their loved one to a nursing home or other assisted living environments. However, many caregivers do not view institutionalization as a viable option for their loved ones due to the unfavorable outcomes that are often associated with this decision. For example, studies have shown that up to half of people with dementia in high income countries like the United States eventually reside in nursing homes or other assisted living facilities and more than two-thirds of care home residents have dementia [28]. However, less than half of the older adults residing in these facilities report good quality of life [28]. Other reasons why older adults and caregivers may be reluctant about nursing homes include media reports about nursing home abuse and costs [28]. Therefore, many caregivers are reluctant or anxious about the prospect of moving their loved ones into these facilities due to low expectations about the care their loved ones would receive and the outcomes associated with that decision. For these reasons, caregivers may elect to keep their loved ones in the community instead. However, this decision may end up exacerbating the challenges reported by many caregivers.

In addition, findings from this study support the growing body of evidence regarding the importance of social support to caregiver and care recipient well-being [29–31]. For example, studies have shown that the caregiver’s ability to cope with the progressing needs of their loved one is dependent on several factors including the caregiver's gender, coping style, social network, and the caregiver’s level of intimacy with their loved one living with dementia [6]. Indeed, availability and accessibility of support from other people, including surrogate caregivers or other paid sources of social support, may act as a buffer in mitigating the negative impact of caregiving on the caregiver’s wellbeing [6]. Consequently, caregivers who report having access to social support report better quality of life and health outcomes compared to caregivers who do not [29–31]. Evidence from the current study supports the viewpoint that caregivers who have a more reliable support system are able to care for their loved ones better because the caregivers themselves are healthier. For these caregivers, feeling healthy and supported is critical to their ability to provide the best possible care for their loved ones living with dementia.

Further, findings from this study shed light on the personal needs of caregivers and why these needs should be taken into consideration when evaluating the care provided to older people living with dementia. Some researchers have referred to family caregivers as the real “victims” of dementia, whose needs are often overlooked in discussions about the impact of dementia [32]. Indeed, some may view this characterization as being unrepresentative of all family caregiver experiences given many caregivers also report positive aspects of caregiving, including a sense of purpose and feelings of fulfillment knowing that one is caring for a loved one in their time of need [13–15]. However, the majority of caregivers’ experiences point to the fact that the energy devoted to caregiving affects the day-to-day lives and well-being of caregivers as they continue to make sacrifices to care for their loved ones even as the level of needs of both the caregivers and care recipient increase over time [3–5, 15, 17, 28]. The sacrifices made by caregivers may constitute progressive losses which impact the caregiver’s emotional, physical, mental, and spiritual health. Over time, the impact of caregiving on the caregiver’s health may be severe enough to render them unable to provide optimal care for their loved one.

Helping caregivers acquire coping skills that can help address their own progressing needs will be important for both the caregiver’s well-being and the care provided to older adults living with dementia. Several caregiver coping strategies have been identified in the literature along with validated instruments to assess their impact [33–35]. These coping strategies include: caregiver mastery, task management strategies, and problem-solving coping. For example, the Perceived Change Index has been used to assess whether a caregiver’s life situation has become worse or improved over the past month, while the Task Management Strategy Index evaluates the extent to which positive caregiving strategies were used to manage activities of daily living dependence and problem behaviors in care recipients [33–35]. These and other instruments provide the opportunity to evaluate the impact of any intervention designed to help caregivers manage the caregiving role.

Overall, the caregivers interviewed in this study express the desire to provide the best possible care to their loved ones living with dementia. They also recognize the importance of maintaining a healthy and balanced quality-of-life, particularly as their loved ones’ condition deteriorates over time. Since caregivers’ needs tend to increase as their loved ones’ condition worsens, efforts must be made to support caregivers in meeting these needs. Caregivers who describe having good support systems seem to be better at coping with the disease progression compared to caregivers with limited support. Social support was identified as an important buffer for caregivers since it provides the opportunity to share some of the burden associated with caregiving. Also, social support contributes to favorable health outcomes for the caregiver including better sleep quality, lower rates of depression, lower burden, and better psychological outcomes [36–38]. As the findings from this study show, these factors improve the caregiver’s own quality of life and can contribute to better care for older people living with dementia.

While this current study was conducted prior to start of the COVID-19 pandemic, it is important to note that restrictions put in place to limit the spread of the virus are exacerbating the challenges faced by caregivers to meet their loved ones’ needs and to keep them safe. Due to social isolation, limitations in the availability of in-home health services, respite care, and other types of informal support, many caregivers are at risk for greater emotional and physical stress [39, 40]. For those caregivers whose loved one may require residential long term support services, this option may not be viable due to increased infection and mortality rates of COVID-19 in these settings [41] and limited access to visitors [42]. Now more than ever, caregivers need psychological and physical support from providers, family, and friends to help preserve their health and wellbeing [39, 43].

### Strengths and Limitations

This study has some limitations. For example, a more diverse sample may yield slightly different themes. For example, studies have shown that family caregivers who are members of a racial or ethnic minority group may face unique challenges when caring for their loved ones [44–46]. Therefore, the lack of racial or ethnic diversity among the study participants could be considered a limitation.

While future studies are needed to further explore the relationship between caregiver QoL and care provided with a greater focus on racial/ethnic identity, type of dementia, and socioeconomic status, the use of both focus group and one-on-one interviews for data collection is an important strength of this study. Also, strategies such as memo keeping, audit trail, and investigator triangulation were used to enhance reliability, validity, and generalizability of the study findings [25–27].

Further, recruitment of participants from two geographic locations is another strength. In addition, interviews and focus groups were conducted in-person, allowing the researchers to observe participants’ body language, facial expression, non-verbal cues, and other relevant reaction.

## Conclusions

Overall, findings from this study show that caregivers perceive a relationship between their own QoL and the quality of care provided to their loved ones living with dementia. Indeed, the decline in cognitive functioning and loss of independence that persons living with dementia experience as their disease progresses over time has implications for both the care recipient and their caregiver’s long-term health and well-being. As this process unfolds, the caregiver may find her/himself being unable to cope with the increasing demands of caring for their loved one. The study highlights the need for the provision of much needed support for caregivers and persons living with dementia.

## Data Availability

The datasets analyzed during the current study are available from the corresponding author (AAH) on reasonable request.

## Abbreviations

QOL: Quality of life
AD: Alzheimer’s disease
START: STrAtegies for RelaTives
NY: New York
IL: Illinois
